# Unearthing Atomic Dynamics in Nanocatalysts

**DOI:** 10.1021/acsami.4c14382

**Published:** 2024-10-25

**Authors:** Antonio J. Martínez-Galera, Rocío Molina-Motos, José M. Gómez-Rodríguez

**Affiliations:** † Departamento de Física de Materiales, Universidad Autónoma de Madrid, Madrid E-28049, Spain; ‡ Condensed Matter Physics Center (IFIMAC), Universidad Autónoma de Madrid, Madrid E-28049, Spain; § Instituto Nicolás Cabrera, Universidad Autónoma de Madrid, Madrid E-28049, Spain; ∥ Departamento de Física de la Materia condensada, Universidad Autónoma de Madrid, Madrid E-28049, Spain

**Keywords:** nanocatalysis, nanoparticles, 2D materials, scanning probe microscopy, surface science

## Abstract

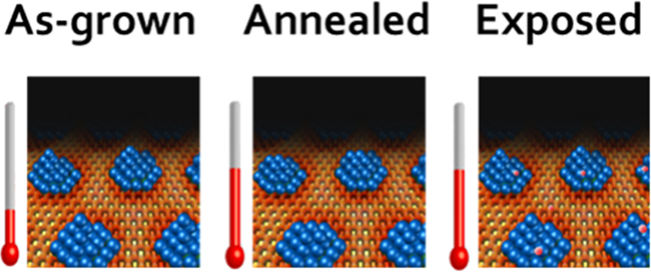

Being able to access the rich atomic-scale phenomenology, which occurs during the reactions pathways, is a pressing need toward the pursued knowledge-based design of more efficient nanocatalysts, precisely tailored atom by atom for each reaction. However, to reach this goal of achieving maximum optimization, it is mandatory, first, to address how exposure to the experimental conditions, which will be needed to activate the processes, affects the internal configuration of the nanoparticles at the atomic level. In particular, the most critical experimental parameter is probably the temperature, which among other unwanted effects can induce nanocatalyst aggregation. This work highlights the high potential of experimental techniques such as the scanning probe microscopies, which are able to investigate matter in real space with atomic resolution, to reach the key challenge in heterogeneous catalysis of achieving access to the atomic-scale processes taking place in the nanocatalysts. Specifically, the phenomenology occurring in a nanoparticle system during annealing is studied with atomic precision by scanning tunneling microscopy. As a result, the existence of an internal atomic restructuring, occurring already at relatively low temperatures, within Ir nanoparticles grown over h-BN/Ru(0001) surfaces is demonstrated. Such restructuration, which reduces the undercoordination of the outer Ir atoms, is expected to have a significant effect on the reactivity of the nanoparticles. Going a step further, an internal restructuring of the nanoparticles during their involvement as catalysts has also been also identified.

## 1 Introduction

Catalysis is an interdisciplinary field which supports more than 90% of the global industrial production^1^ and different research areas as green energy production,^2^ fuel development,^3^ medicine,^4,5^ and environmental science,^6^ among others.^7,8^ For this reason, the development of ever more efficient nanocatalysts is a major priority in the scientific community. It is well-known that catalysts undergo different changes as a consequence of their interaction with the reactants and byproducts as well as due to their exposure to experimental conditions, as for instance the temperature, required for each reaction.^9−17^ These changes affect their internal structure, modifying the undercoordination of the outer atoms and the available adsorption sites for the reactants.^10,14−16^ Hence, such alterations occurring along the reaction pathway compromise the long-term performance of the catalysts. Therefore, to achieve the ultimate in knowledge-based rational design, it is necessary to precisely identify these changes by disentangling the effects associated with the exposure of the nanocatalysts to the experimental conditions needed for the reaction from those linked to their interaction with the reactants and byproducts. It will ease the highly pursued atom by atom design of more efficient nanocatalysts specifically tailored for each reaction.

A longstanding problem hindering a faster advance in knowledge-based nanocatalysts design is the lack of precise access to the rich variety of processes occurring at the atomic scale.^18−21^ It is, in part, due to the fact that usually the internal structure of the nanocatalysts is investigated by using experimental techniques providing averaging information. However, in order to access the ample atomistic-level phenomenology occurring along each pathway, it is necessary to investigate the processes using techniques providing local information with atomic accuracy. To this end, interesting candidates are the scanning probe microscopies (SPMs), which are able to study directly in real space the local properties of nanomaterials with atomic resolution. On the other hand, model catalysts precisely tailored and characterized at the atomic scale are rarely employed in heterogeneous catalysis research, which hinders a more precise interpretation of the experiment’s outcome. In this regard, two-dimensional (2D) materials such as graphene and hexagonal boron nitride (h-BN), which can be grown on a wide variety of supports,^22−35^ have proven to be excellent templates for the development of ordered arrays of atomically tailored nanoparticles.^36−43,34,44−46^

Here, an atomic-scale study of the internal changes taking place upon annealing within metal nanoparticles is performed by using scanning tunneling microscopy (STM) as the main characterization technique. The nanomaterial used here as a model nanocatalyst system comprises a one-atom-thick layer of h-BN grown on Ru(0001) as the support, and the nanoparticles grown on top, which are made out of Ir, an outstanding Pt group catalyst. The so-obtained nanoparticles are found to exhibit a well-defined crystalline structure, and their average size can be tuned with precision through the Ir amount deposited on the h-BN/Ru(0001) surfaces. This investigation has allowed us to determine the existence of atomic-scale modifications in the nanoparticles during the exposure at high temperatures. This internal reconfiguration involves changes in the undercoordination of the outer Ir atoms comprising the nanoparticles, which, in turn, is expected to have a clear impact on their catalytic activity.

## 2 Experimental Section

Experiments were performed in an ultrahigh-vacuum (UHV) system, equipped with a home-built variable temperature scanning tunneling microscope (VT-STM) and combined low-energy electron diffraction (LEED)/Auger electron spectroscopy (AES) optics as the main characterization instruments.^47^

Ru(0001) surfaces were prepared by Ar^+^ sputtering at 1 keV with the Ru single crystal at 850 °C, followed by annealing at the same temperature at an O_2_ partial pressure of 2 × 10^–6^ Torr and subsequent flash annealing at 1100 °C without supplying O_2_. The growth of one-atom thick layers of h-BN on the Ru(0001) surfaces was carried out by chemical vapor deposition using borazine (B_3_H_6_N_3_) as precursor. In more detail, the Ru(0001) surfaces kept at 800 °C were exposed at a partial pressure of borazine of 2.5 × 10^–8^ Torr during 35 min. Nanoparticle growth was accomplished by Ir deposition on the h-BN/Ru(0001) surfaces at RT from a current heated filament made of that element. The deposition rate has been accurately calibrated as a function of the filament current from STM images acquired after Ir deposition on an Ir(111) surface. Therefore, the Ir coverages indicated in this article are referenced to the atomic density of the (111) planes of the crystal structure of Ir, meaning that 1 ML correspond to 1.6 × 10^19^ Ir atoms deposited per 1 m^2^. The Ir coverage employed for each data set is indicated in the respective figure captions. After nanoparticle growth, the atomic dynamics following annealing have been studied, and the corresponding sample temperatures are also specified in the respective figure captions. Accordingly, the atomic dynamics occurring along the dissociative chemisorption of O_2_ on the nanoparticles at high temperatures have been studied as well. Both the sample temperature and the O_2_ exposure amount are provided in the corresponding figure captions.

STM data acquisition was performed by using WSxM software,^48^ with the bias voltage applied to the sample. The apparent height histograms were elaborated from the measured STM images by means of a source code developed in a previous work.^44^ For these histograms, a total of 67526 nanoparticles were evaluated.

## 3 Results and Discussion

**Figure 1:**
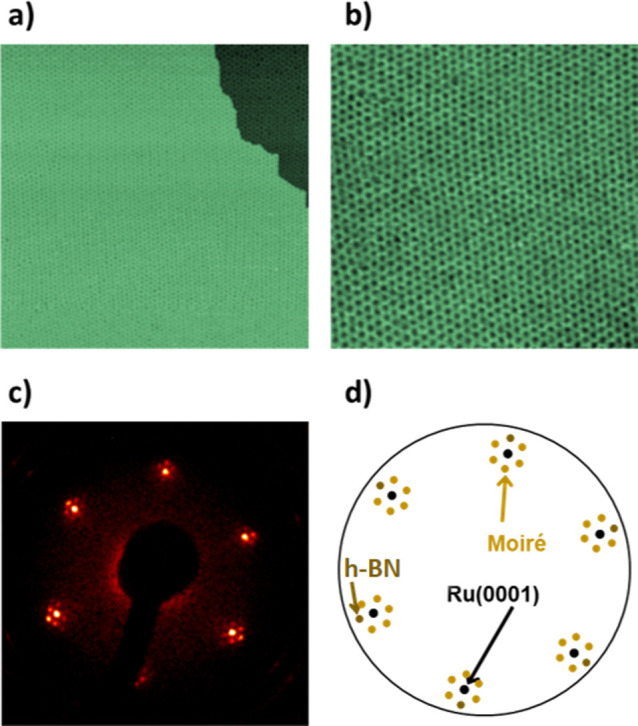
Structural properties of h-BN/Ru(0001). (a) Overview STM topograph displaying the sample morphology. (b) STM image providing a magnified view of the moiré pattern characteristic of h-BN/Ru(0001) surfaces. (c) LEED pattern acquired at an energy of 45 eV on a h-BN/Ru(0001) surface. (d) Schematic representation of the LEED pattern showed in (c). The spots associated with the atomic periodicity of the Ru(0001) surface are represented in black, while those associated with the atomic arrangement of the h-BN layer are represented in brown. The satellite spots represented in light brown correspond to the periodicity of the moiré pattern, which is aligned with the atomic arrangements of both Ru(0001) and h-BN. Tunneling parameters: (a) *I*_T_ = 3 nA, *V*_s_ = +0.6 V, size = 200 × 200 nm^2^; (b) *I*_T_ = 1.3 nA, *V*_s_ = +1.35 V, size = 100 × 100 nm^2^.

As a reference, Figure 1 summarizes the most representative structural features of the h-BN/Ru(0001) surfaces. In more detail, in consistency with the available literature on this h-BN/metal interface,^49^Figure 1a shows a large-scale STM image displaying a unique moiré pattern with a periodicity of 3.2 nm. This pattern, which arises from the superposition of the atomic arrangements of the metal surface and the 2D material, will act as a template for the subsequent growth of the nanoparticle networks. A closer view of a surface region, showing the so-called pores (darker dots) comprising the moiré structure, is displayed in Figure 1b. Likewise, a LEED pattern revealing the structure of the moiré superstructure in reciprocal space is provided in Figure 1c.

To ease the interpretation of the moiré pattern, Figure 1d shows a schematic representation indicating the origin of the respective diffraction spots. It is inferred that the atomic lattice of the Ru(0001) surface is aligned with the atomic arrangement of h-BN and with the moiré pattern. The absence of arcs associated with the atomic periodicity of h-BN demonstrates that there is no other rotational domain, consistent with the observation in STM images of a unique moiré pattern along the h-BN/Ru(0001) surfaces.

**Figure 2:**
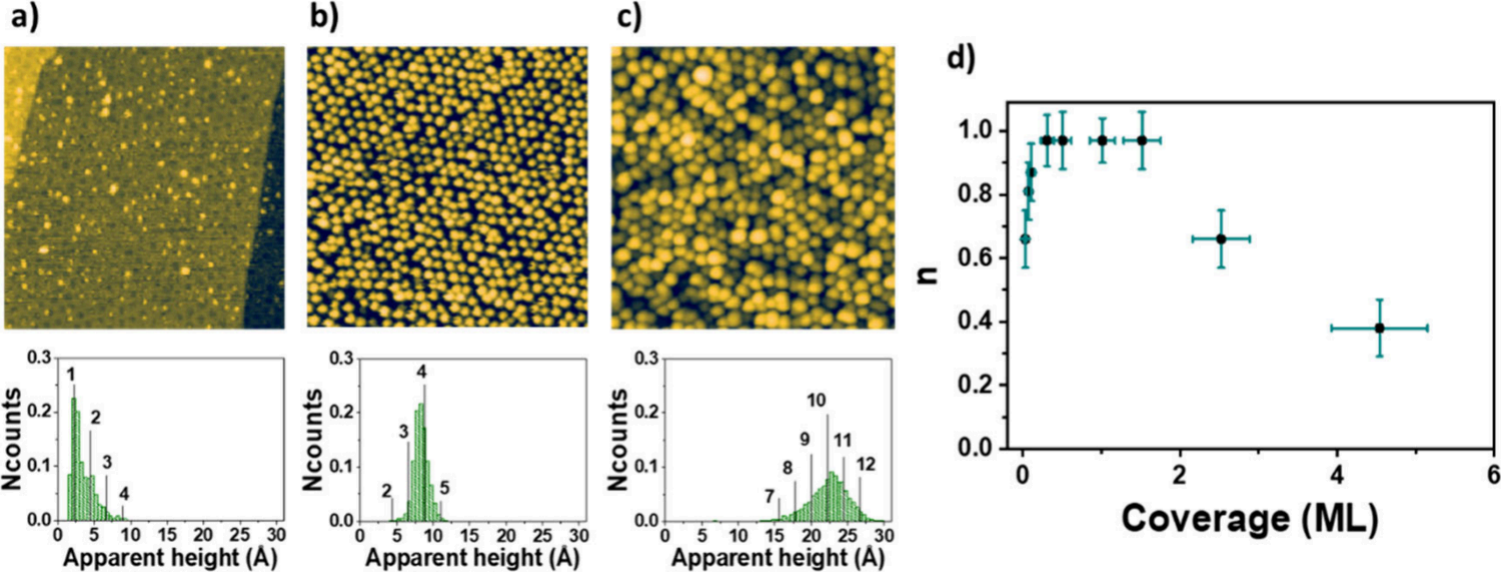
Evolution of the spatial distribution and the internal structure of the nanoparticles with the Ir amount deposited on h-BN/Ru(0001). (a–c) STM images illustrating the spatial distribution (top panels) and apparent height histograms (lower panels) providing information about the internal structure of the Ir aggregates formed after 0.03 ± 0.01, 0.31 ± 0.08, and 4.5 ± 0.6 ML Ir deposition on h-BN/Ru(0001). Tunneling parameters: *V*_s_ = +2.4 V, *I*_T_ = 0.34 nA; size: 70 × 70 nm^2^ for all the STM images. The vertical lines in the histograms represent the heights, which would correspond to integer multiples of the distance between consecutive (111) planes of Ir. The corresponding integer multiple is labeled close to each line. (d) Evolution of the average number of nanoparticles per moiré pore *n* with the Ir coverage.

Once the h-BN/Ru(0001) supports were obtained, the next step was to try to use them as templates for the growth of nanoparticles, tailored at the atomic scale, with the potential to be used as model catalysts. Figure 2 shows the evolution of both the nanoparticle size and their spatial distribution over the h-BN/Ru(0001) surface with the deposited amount of Ir. As observed in the upper panel of Figure 2a, during the initial stages of the growth, nanoparticles are found on the moiré pores, although not all of them are occupied by an Ir aggregate. For larger coverages, the spatial distribution of nanoparticles follows the regular pattern dictated by the moiré superperiodicity, reaching an almost complete occupancy of all the pores (see the upper panel of Figure 2b). In contrast, for larger amounts of Ir, the density of the nanoparticles starts to decrease due to coalescence. The respective apparent height distributions provided in the lower panels show a gradual shift toward larger values with the deposited amount and a coincidence between the peaks’ positions and the spacing between consecutive (111) planes of the crystal structure of Ir.

The spatial distribution observed in Figure 2 shows the existence of three growth regimes, which have been also found in other nanoparticle systems formed on 2D materials.^36,44^ In the first regime, both the density of nanoparticles and their apparent heights increase with the amount of Ir deposited on the h-BN/Ru(0001) surface until almost all moiré pores become occupied. Then, in a second stage, nanoparticle sizes increase with the deposited amount, while their density over the support does not change, remaining close to one Ir aggregate per moiré pore. Upon further Ir deposition, the next phase is reached, in which nanoparticle size increases with Ir coverage, but the density of atomic aggregates over the h-BN/Ru(0001) is reduced due to coalescence. These three stages are also clearly distinguished in the plot analyzing the evolution of the moiré pores occupation with increasing the Ir coverage shown in Figure 2d.

As mentioned, the existence of these three regimes has been previously found on other nanoparticle systems. Nevertheless, significant differences become apparent when comparing with the growth of Ir nanoparticles on the analogue h-BN/Rh(111) surfaces.^44^ Specifically, larger apparent heights values are obtained for the Ir nanoparticles grown on h-BN/Ru(0001) compared to these formed on h-BN/Rh(111),^44^ for the same coverages. In principle, a possible explanation could be that the lateral dimensions of the atomic layers comprising the nanoparticles must be smaller on h-BN/Ru(0001) for the same amounts of Ir. However, nanoparticle coalescence does not start at larger coverages than for the case of the Ir aggregates grown on h-BN/Rh(111) (see Figure 2). Other possibility could be an earlier beginning of the growth of the topmost layers before the atomic Ir planes below become complete. It could result in a higher undercoordination of Ir atoms in the nanoparticles and hence in a higher reactivity.

**Figure 3:**
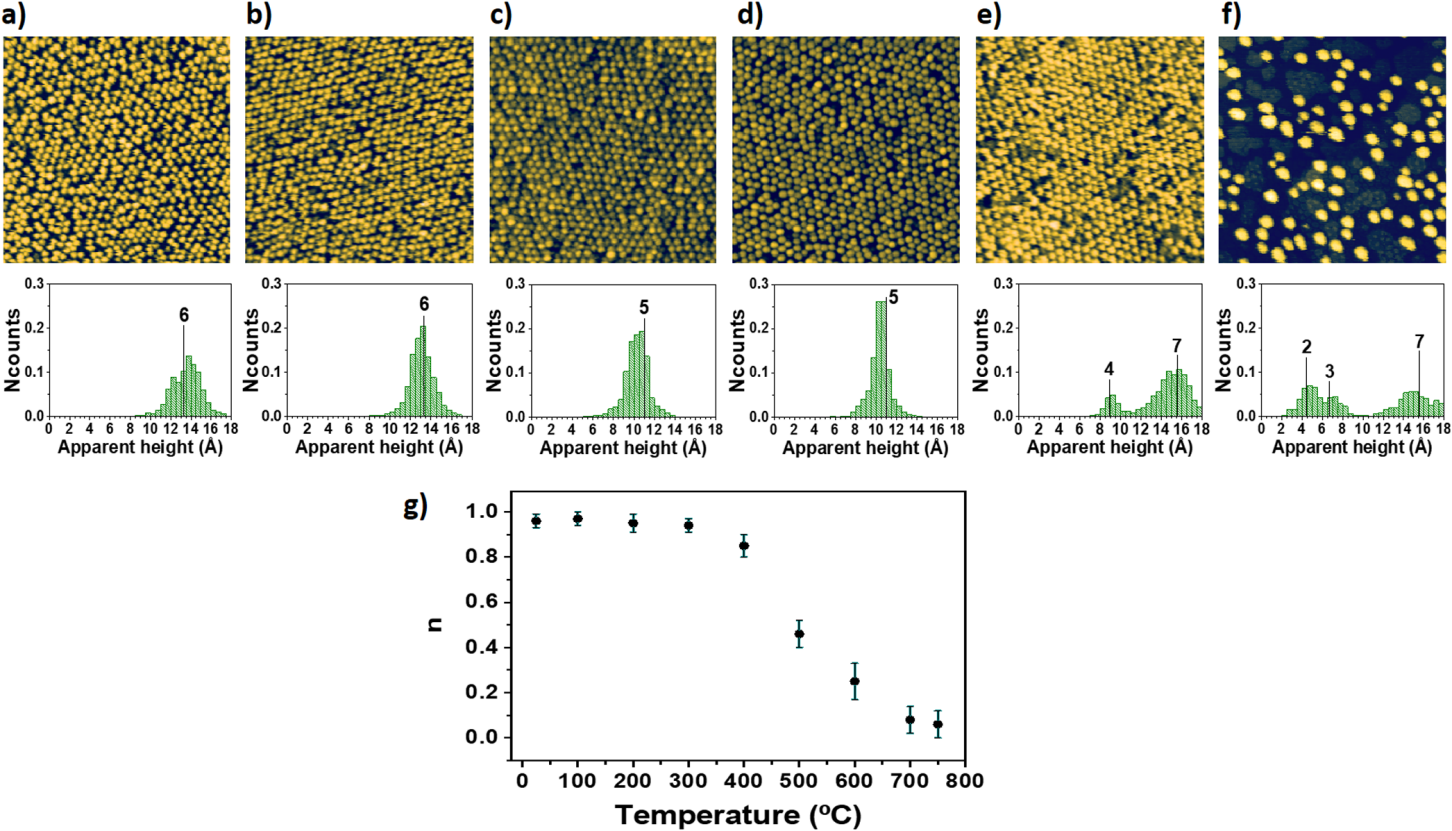
Evolution of the spatial distribution and the internal structure of the Ir nanoparticles with the annealing temperature. (a–f) STM images (upper panels) and apparent height distributions (lower panels) of (a) nanoparticles grown by depositing 0.9 ± 0.1 ML of Ir over h-BN/Ru(0001) surfaces and (b–f) subsequent annealing for 300 s at increasingly growing temperatures in steps of 100 °C. Annealing step: (b) 100, (c) 200, (d) 300, (e) 400, and (f) 600 °C. Tunneling parameters: *V*_s_ = +2.4 V, *I*_T_ = 0.34 nA; size: 80 × 80 nm^2^ for all the STM images. The vertical lines outlined in the histograms represent the heights, which would correspond to integer multiples of the distance between consecutive (111) planes of Ir. (g) Dependency of the average number of nanoparticles per moiré pore *n* with the annealing temperature.

A crucial aspect for a nanoparticle system to be considered as a potential catalyst is its thermal stability. Figure 3 shows the evolution with the annealing temperature of Ir nanoparticles grown on an h-BN/Ru(0001) support. As observed, the spatial distribution of the nanoparticle system is kept intact after stepwise annealing at increasing temperatures until 300 °C. However, a deep analysis of the internal structure, through apparent height histograms, demonstrates the existence of changes in the atomic-scale configuration of the nanoparticles already at this temperature range. To understand these changes, it is worth nothing to mention first, as a reference, that the as-grown nanoparticles were mainly composed of six atomic planes, showing a structure analogue to that of the (111) Ir facets, although aggregates with five and seven layers were also present at a significant extent. Then, by annealing at 100 °C for 300 s, the apparent height distribution is narrowed around values corresponding to six Ir(111) planes. Further annealing at higher temperatures until 300 °C resulted in a reduction in the average number of atomic layers in the nanoparticles. Lastly, at higher annealing temperatures the resulting nanoparticle coalescence gives rise to a decrease in the average occupation of the h-BN/Ru(0001) pores (see also Figure 3g). Additionally, further atomic-scale restructuration of nanoparticles occurs at these temperatures as inferred by a broadening of the apparent height distribution. Both the reduction in the occupation number and the apparent height broadening are gradually accentuated after ensuing stepwise annealing at higher temperatures.

The changes in the atomic-scale configuration of the nanoparticles could be interpreted as follows. Nanoparticles formation could occur according to a Volmer–Weber growth mode, in which their outermost layers would start to grow before the preceding ones are completed. After annealing, atoms placed in the upper layers of the nanoparticles could diffuse to occupy empty positions in the lower ones. This restructuring would directly impact the undercoordination of the outer Ir atoms in the nanoparticles, leading to more complete layers and, as a result, a reduction in the number of Ir atoms that lack neighboring ones. It, in turn, is expected to have an impact on the performance of these Ir atomic aggregates as potential catalysts. Here, it is convenient to note that, in general, for being quasi-zero-dimensional objects, nanoparticles exhibit catalytic activities enhanced with respect to bulk materials due to the higher undercoordination of the outer atoms. Interestingly, these changes in the atomic-scale configuration of nanoparticles are different from those reported over h-BN/Rh(111) surfaces,^44^ suggesting an influence of the metal support below the h-BN layer. On the other hand, the reduction of the occupation number observed after annealing at temperatures of around 400 °C and higher could be interpreted as the result of mass transport between the h-BN/Ru(0001) pores, giving rise to Ostwald ripening. This effect results in the formation of new nanoparticles with larger sizes and varying undercoordination of their outer Ir atoms and hence with an expected dissimilar catalytic activity.

**Figure 4:**
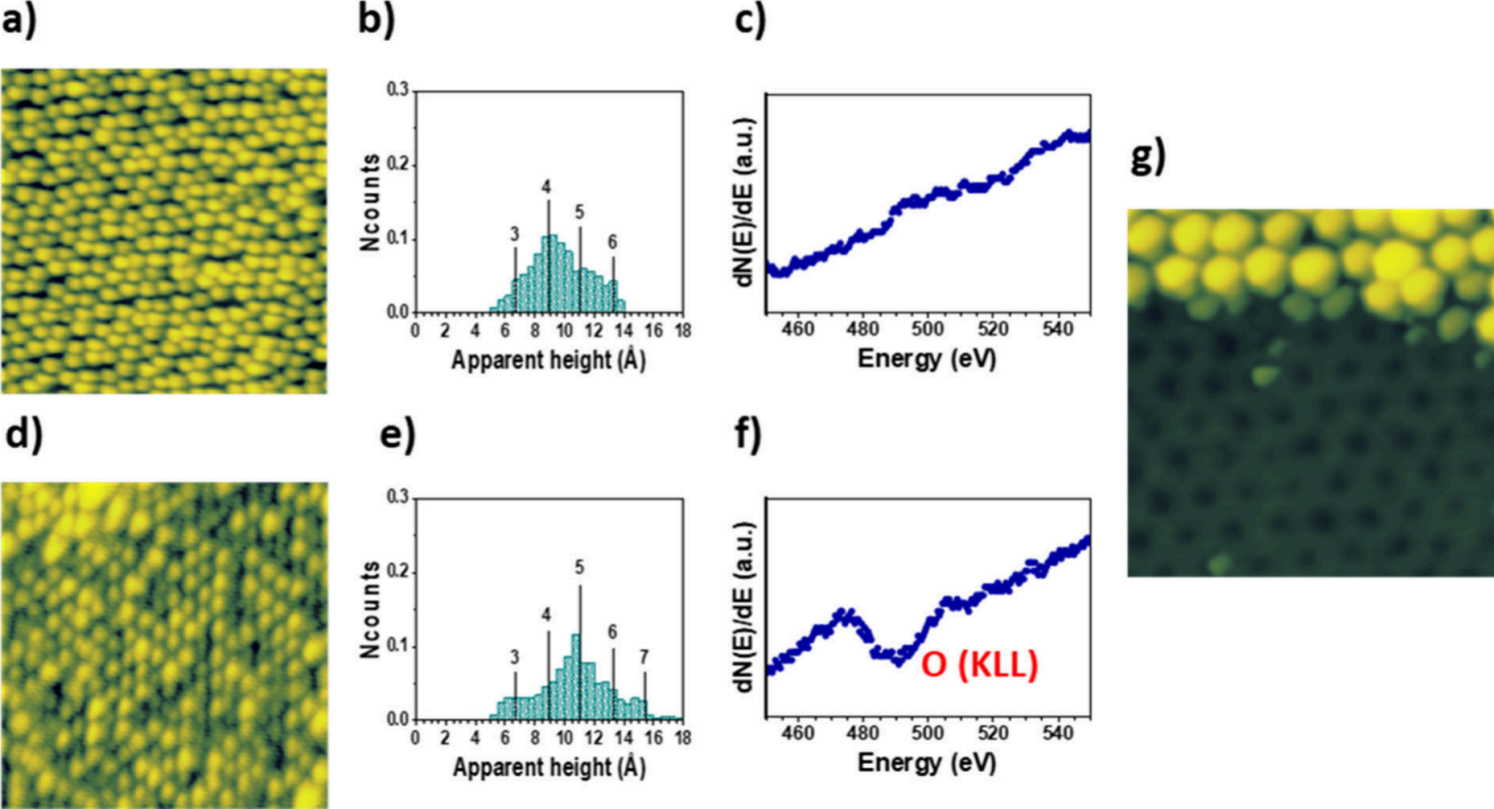
Spatial distribution over the h-BN surface and internal structure of the Ir nanoparticles after their involvement as nanocatalysts in the dissociative chemisorption of oxygen. (a) STM image, (b) apparent height histogram, and (c) AES spectrum, obtained in a sample resulting from 0.81 ± 0.09 ML Ir deposition on a h-BN/Ru(0001) surface; (d) STM image, (e) apparent height histogram, and (f) AES spectrum obtained after 3 × 10^4^ langmuir O_2_ exposure, at a partial pressure of 0.1 mTorr, the sample analyzed in (a–c) at a temperature of 300 °C. (g) STM image showing the underlying h-BN/Ru(0001) surface after the removal, by using the STM tip, of the Ir nanoparticles in a region of the sample analyzed in (d–f). Tunneling parameters: (a) *I*_T_ = 18 pA, *V*_s_ = −2.4 V, size = 50 × 50 nm^2^; (d) *I*_T_ = 18 pA, *V*_s_ = −2.4 V, size = 50 × 50 nm^2^; (g) *I*_T_ = 0.43 nA, *V*_s_ = −1.3 V, size = 25 × 25 nm^2^.

Once the effects of annealing have been addressed, the atomic dynamics within the nanoparticles associated with their involvement as catalysts in a reaction will be investigated. To this end, the dissociative chemisorption of molecular oxygen has been considered as a model reaction, which is a key step in most industrial processes based on oxidation.^50,51^ In this light, it is interesting to highlight that oxidation reactions support around one-third of the chemical production and play a significant role in important applications.^7,52^Figure 4 summarizes the experimental findings of this study. The comparison of Figures 4a and 4d demonstrates that after 3 × 10^4^ langmuir O_2_ exposure at a partial pressure of 0.1 mTorr and a sample temperature of 300 °C, the Ir nanoparticle network is preserved. Concerning the internal structure of nanoparticles, O_2_ exposure at these conditions causes a broadening of the size distribution, which is also shifted toward larger values (compare histograms provided in Figures 4b,e). The presence of oxygen on the samples after their exposure to O_2_ under the above-mentioned experimental conditions is confirmed by Auger electron spectroscopy (compare spectra of Figures 4c and 4f).

To interpret the results summarized in Figure 4, it is important to note that the presence of oxygen after O_2_ exposure under the above-mentioned conditions is a strong indication of dissociative chemisorption of oxygen. In this regard, Figure 4g displays an STM image showing the supporting h-BN/Ru(0001) surface after the removal, by using the STM tip, of the Ir nanoparticles at a certain region of a sample exposed to O_2_ under the mentioned conditions. The structural quality of the moiré superstructure demonstrates the absence of significant oxidation of the h-BN layer, hence suggesting that oxygen must be adsorbed mainly on the Ir nanoparticles. Nevertheless, it should be mentioned here that to keep their molecular form, the O_2_ molecules only can interact with the Ir atoms of the nanoparticles through dispersive interactions, which would not be expected not to lead to desorption at 300 °C. Therefore, the most likely scenario is that in which oxygen is adsorbed on the Ir nanoparticles due to the dissociative chemisorption of O_2_ molecules. This picture is also consistent with the atomic-scale variations taking place in the Ir nanoparticles (compare histograms provided in Figures 4b,e), which will be discussed in the next paragraph.

The dissociative chemisorption of oxygen is known to cause stress in most metal surfaces.^53−55^ It arises from the ionic character of the binding between O atoms and those of the metal surface, the resulting charge transfer between them, and the consequent electrostatic repulsion between charges of the same type. As a result, to reduce the stress, metal atoms in the surface are often pushed out, forming overlayer islands with the same atomic arrangement as in the terraces of the surface.^53−55^ The shift toward larger values of the apparent height distribution observed in the histogram displayed in Figure 4e, whose peaks still coincide with integer multiples of the distance between (111) planes of Ir, is consistent with this pìcture. It is an indication that the above-described phenomenology reported in metal surfaces could also occur in the Ir nanoparticles. Moreover, this effect is known to cause roughening in the metal surfaces due to the presence of the overlayer islands,^53−55^ which is also consistent with the broadening of the apparent height histograms observed here for the Ir nanoparticles.

**Figure 5:**
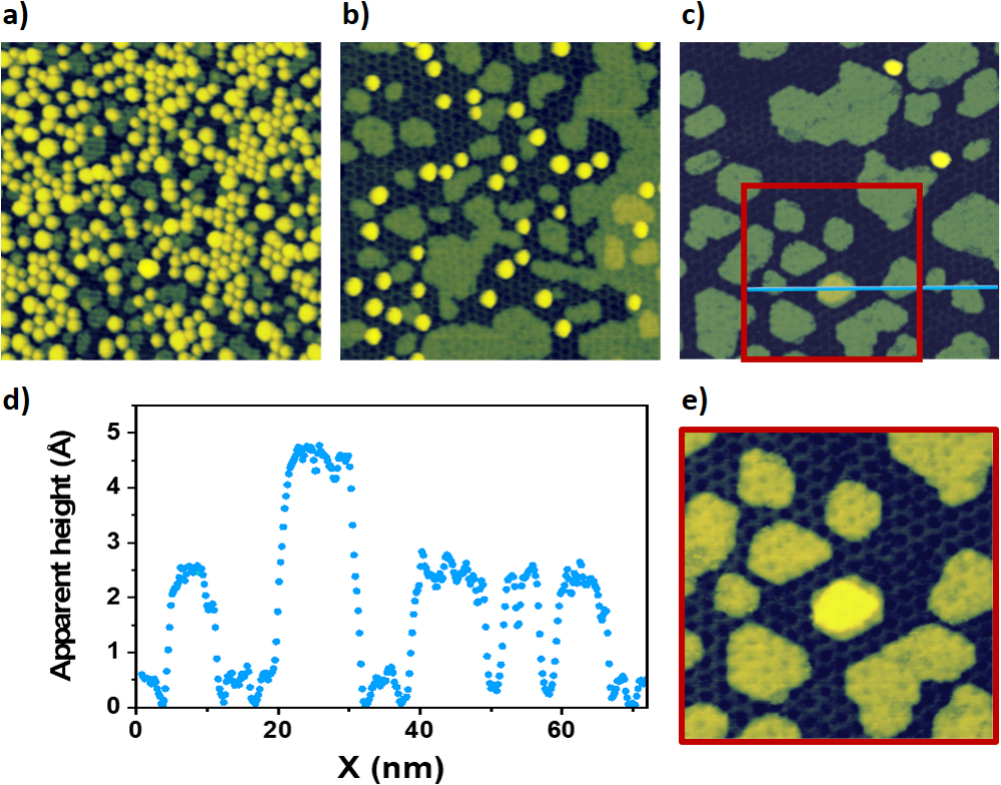
Ir intercalation between the h-BN monolayer and the underlying Ru(0001) surface by annealing the Ir nanoparticle systems at temperatures above 400 °C. (a–c) STM images acquired on the sample analyzed in Figure 3 after the annealing steps of (a) 500, (b) 700, and (c) 750 °C (for this last step sample temperature was held during 3900 s). (d) Apparent height profile along the blue line highlighted in (c). (e) STM image acquired in the red square outlined in (c). Tunneling parameters: (a, b) *V*_s_ = +2.4 V, *I*_T_ = 0.35 nA, size: 90 × 90 nm^2^; (c) *V*_s_ = +1.64 V, *I*_T_ = 0.35 nA, size: 90 × 90 nm^2^; (e) *V*_s_ = +1.64 V, *I*_T_ = 0.35 nA; size: 50 × 50 nm^2^.

An interesting effect observed after annealing at temperatures higher than 400 °C without oxygen exposure is the development of flat islands, which are found scattered around the surface in sample regions between the nanoparticles. As observed in Figure 5, these islands occupy a larger fraction of the sample as the annealing temperature increases. For the sample analyzed in Figure 5, these islands occupy about 60% of the surface at the end of the annealing sequence. The apparent height of these islands is around 2.5 Å, although for a few of them, it can be roughly double (see Figure 5d). The edges of these islands are aligned with the moiré superstructure, which is faintly observed superimposed over the islands, as observed in Figure 5e.

The fact that these islands cover a larger fraction of the surface as the number of nanoparticles decreases is strong evidence that they must be composed of Ir. In addition, considering their apparent heights, it is reasonable to suggest that they are mostly monolayer islands, except those whose apparent height is double, which are likely bilayer islands. This picture is consistent with the initial Ir amount of 0.9 ± 0.1 ML deposited in this caseover the h-BN/Ru(0001) surface for nanoparticle growth, given the fact that at the end of the annealing sequence, these islands span around 60% of the sample. Therefore, the most plausible scenario could be a pseudomorphic growth of these islands over the Ru(0001) surface below the h-BN monolayer. The difference between their extension of 60% over the sample and the 90 ± 10%, which would be expected for a pseudomorphic growth after 0.9 ± 0.1 ML deposition, could be explained by the presence of still a few Ir nanoparticles and bilayer islands along the sample at the end of the annealing sequence as well as by a possible Ir diffusion into the subsurface of the Ru(0001) crystal. The formation of these islands over the h-BN/Ru(0001) surface as an alternative explanation can be excluded from the experiments presented in Figure S1 of the Supporting Information. These experiments demonstrate the growth of nanoparticles instead of flat islands, covering the whole sample after subsequent Ir deposition following the completion of the annealing sequence. The resulting nanoparticle network has the periodicity of the moiré pattern.

## 4 Conclusions

This work demonstrates how STM can provide access to the atomic-scale changes occurring on nanoparticles during annealing as well as during their involvement as catalysts. A precise picture of the internal restructuring of Ir nanoparticles supported on h-BN/Ru(0001) surfaces as the temperature is raised has been achieved. Specifically, nanoparticle growth proceeds via a Volmer–Weber scheme in such a way that the upper atomic layers comprising the aggregates start to grow before the lower ones are completed. After annealing at temperatures below 400 °C, the experimental findings are consistent with a thermal activated motion of Ir atoms from the upper atomic planes toward the lower ones, hence reducing nanoparticle height. As these internal changes are inherently accompanied by changes in the undercoordination of the atoms on the surface of the nanoparticles, this effect must imply changes in the catalytic properties. For this reason, in order to perform well-controlled nanocatalysis studies on these nanoparticles, it is first necessary to address this effect prior to studying the interaction of these atomic aggregates with the reactants and their byproducts. It is noteworthy to mention that these changes can be demonstrated only by using experimental techniques providing high resolution in real space as the SPMs. After annealing at temperatures over 400 °C, nanoparticles start to decay due to Ir intercalation at the h-BN/Ru(0001) interface. Additionally, during their involvement as catalysts in the dissociative chemisorption of oxygen, the Ir nanoparticles undergo internal transformations, giving rise to a broader apparent height distribution which, in addition, is shifted toward larger values. It is consistent with a scenario where the stress in the outer atomic layer of the nanoparticles, induced by oxygen adsorption, is relieved by pushing out Ir atoms to form an additional overlayer. This work highlights the high potential of the SPMs to contribute to a more accurate knowledge-based rational design of nanocatalysts by providing access to the atomic-scale processes occurring along the reactions.

## Supporting Information Available

STM characterization of nanoparticles grown after partial Ir intercalation and AES characterization of the Ir nanoparticles grown on h-BN/Ru(0001) (PDF)

## References

[ref1] Armor J. N. A History of Industrial Catalysis. Catal. Today 2011, 163, 3–9. 10.1016/j.cattod.2009.11.019.

[ref2] Steele B. C. H.; Heinzel A. Materials for Fuel-Cell Technologies. Nature 2001, 414, 345–352. 10.1038/35104620. 11713541

[ref3] Primo A.; Garcia H. Zeolites as Catalysts in Oil Refining. Chem. Soc. Rev. 2014, 43, 7548–7561. 10.1039/C3CS60394F. 24671148

[ref4] Johnson N. B.; Lennon I. C.; Moran P. H.; Ramsden J. A. Industrial-Scale Synthesis and Applications of Asymmetric Hydrogenation Catalysts. Acc. Chem. Res. 2007, 40, 1291–1299. 10.1021/ar700114k. 17803270

[ref5] Savile C. K.; Janey J. M.; Mundorff E. C.; Moore J. C.; Tam S.; Jarvis W. R.; Colbeck J. C.; Krebber A.; Fleitz F. J.; Brands J.; Devine P. N.; Huisman G. W.; Hughes G. J. Biocatalytic Asymmetric Synthesis of Chiral Amines from Ketones Applied to Sitagliptin Manufacture. Science 2010, 329, 305–309. 10.1126/science.1188934. 20558668

[ref6] Chong M. N.; Jin B.; Chow C. W. K.; Saint C. Recent Developments in Photocatalytic Water Treatment Technology: A Review. Water Res. 2010, 44, 2997–3027. 10.1016/j.watres.2010.02.039. 20378145

[ref7] Valange S.; Vedrine J. C. General and Prospective Views on Oxidation Reactions in Heterogeneous Catalysis. Catalysts 2018, 8, 483 10.3390/catal8100483.

[ref8] Nanoscale Engineering for Sustainable Catalysis. Nat. Nanotechnol. 2021, 16, 117–117. 10.1038/s41565-021-00862-y. 33568806

[ref9] Lu J.; Fu B.; Kung M. C.; Xiao G.; Elam J. W.; Kung H. H.; Stair P. C. Coking- and Sintering-Resistant Palladium Catalysts Achieved through Atomic Layer Deposition. Science 2012, 335, 1205–1208. 10.1126/science.1212906. 22403386

[ref10] Vendelbo S. B.; Elkjaer C. F.; Falsig H.; Puspitasari I.; Dona P.; Mele L.; Morana B.; Nelissen B. J.; van Rijn R.; Creemer J. F.; Kooyman P. J.; Helveg S. Visualization of Oscillatory Behaviour of Pt Nanoparticles Catalysing Co Oxidation. Nat. Mater. 2014, 13, 884–890. 10.1038/nmat4033. 25038730

[ref11] Chi M.; Wang C.; Lei Y.; Wang G.; Li D.; More K. L.; Lupini A.; Allard L. F.; Markovic N. M.; Stamenkovic V. R. Surface Faceting and Elemental Diffusion Behaviour at Atomic Scale for Alloy Nanoparticles During in Situ Annealing. Nat. Commun. 2015, 6, 8925 10.1038/ncomms9925. 26576477 PMC4673855

[ref12] Gao J.; Liu Q.; Gu F.; Liu B.; Zhong Z.; Su F. Recent Advances in Methanation Catalysts for the Production of Synthetic Natural Gas. Rsc Advances 2015, 5, 22759–22776. 10.1039/C4RA16114A.

[ref13] Al Abdulghani A. J.; Park J.-H.; Kozlov S. M.; Kang D.-C.; AlSabban B.; Pedireddy S.; Aguilar-Tapia A.; Ould-Chikh S.; Hazemann J.-L.; Basset J.-M.; Cavallo L.; Takanabe K. Methane Dry Reforming on Supported Cobalt Nanoparticles Promoted by Boron. J. Catal. 2020, 392, 126–134. 10.1016/j.jcat.2020.09.015.

[ref14] Liu L.; Corma A. Evolution of Isolated Atoms and Clusters in Catalysis. Trends in Chemistry 2020, 2, 383–400. 10.1016/j.trechm.2020.02.003.

[ref15] Muravev V.; Spezzati G.; Su Y.-Q.; Parastaev A.; Chiang F.-K.; Longo A.; Escudero C.; Kosinov N.; Hensen E. J. M. Interface Dynamics of Pd-Ceo_2_ Single-Atom Catalysts During Co Oxidation. Nature Catalysis 2021, 4, 469–478. 10.1038/s41929-021-00621-1.

[ref16] Ghosh T.; Arce-Ramos J. M.; Li W.-Q.; Yan H.; Chee S. W.; Genest A.; Mirsaidov U. Periodic Structural Changes in Pd Nanoparticles During Oscillatory Co Oxidation Reaction. Nat. Commun. 2022, 13, 6176 10.1038/s41467-022-33304-x. 36261440 PMC9582216

[ref17] Amirbeigiarab R.; Tian J.; Herzog A.; Qiu C.; Bergmann A.; Cuenya B. R.; Magnussen O. M. Atomic-Scale Surface Restructuring of Copper Electrodes under Co_2_ Electroreduction Conditions. Nature Catalysis 2023, 6, 837–846. 10.1038/s41929-023-01009-z.

[ref18] Jin R. The Impacts of Nanotechnology on Catalysis by Precious Metal Nanoparticles. Nanotechnol. Rev. 2012, 1, 31–56. 10.1515/ntrev-2011-0003.

[ref19] Cao K.; Zoberbier T.; Biskupek J.; Botos A.; McSweeney R. L.; Kurtoglu A.; Stoppiello C. T.; Markevich A. V.; Besley E.; Chamberlain T. W.; Kaiser U.; Khlobystov A. N. Comparison of Atomic Scale Dynamics for the Middle and Late Transition Metal Nanocatalysts. Nat. Commun. 2018, 9, 3382 10.1038/s41467-018-05831-z. 30139935 PMC6107508

[ref20] Rong H.; Ji S.; Zhang J.; Wang D.; Li Y. Synthetic Strategies of Supported Atomic Clusters for Heterogeneous Catalysis. Nat. Commun. 2020, 11, 5884 10.1038/s41467-020-19571-6. 33208740 PMC7674434

[ref21] Zachman M. J.; Fung V.; Polo-Garzon F.; Cao S.; Moon J.; Huang Z.; Jiang D.-e.; Wu Z.; Chi M. Measuring and Directing Charge Transfer in Heterogenous Catalysts. Nat. Commun. 2022, 13, 3253 10.1038/s41467-022-30923-2. 35668115 PMC9170698

[ref22] Oshima C.; Nagashima A. Ultra-Thin Epitaxial Films of Graphite and Hexagonal Boron Nitride on Solid Surfaces. J. Phys.-Condes. Matter 1997, 9, 1–20. 10.1088/0953-8984/9/1/004.

[ref23] Corso M.; Auwarter W.; Muntwiler M.; Tamai A.; Greber T.; Osterwalder J. Boron Nitride Nanomesh. Science 2004, 303, 217–220. 10.1126/science.1091979. 14716010

[ref24] Preobrajenski A. B.; Nesterov M. A.; Ng M. L.; Vinogradov A. S.; Martensson N. Monolayer H-Bn on Lattice-Mismatched Metal Surfaces: On the Formation of the Nanomesh. Chem. Phys. Lett. 2007, 446, 119–123. 10.1016/j.cplett.2007.08.028.

[ref25] Cavar E.; Westerstrom R.; Mikkelsen A.; Lundgren E.; Vinogradov A. S.; Ng M. L.; Preobrajenski A. B.; Zakharov A. A.; Martensson N. A Single H-Bn Layer on Pt(111). Surf. Sci. 2008, 602, 1722–1726. 10.1016/j.susc.2008.03.008.

[ref26] Coraux J.; N’Diaye A. T.; Busse C.; Michely T. Structural Coherency of Graphene on Ir(111). Nano Lett. 2008, 8, 565–570. 10.1021/nl0728874. 18189442

[ref27] N’Diaye A. T.; Coraux J.; Plasa T. N.; Busse C.; Michely T. Structure of Epitaxial Graphene on Ir(111). New J. Phys. 2008, 10, 043033 10.1088/1367-2630/10/4/043033.

[ref28] Vazquez de Parga A. L.; Calleja F.; Borca B.; Passeggi M. C. G. Jr.; Hinarejos J. J.; Guinea F.; Miranda R. Periodically Rippled Graphene: Growth and Spatially Resolved Electronic Structure. Phys. Rev. Lett. 2008, 100, 056807 10.1103/PhysRevLett.100.056807. 18352412

[ref29] Wintterlin J.; Bocquet M. L. Graphene on Metal Surfaces. Surf. Sci. 2009, 603, 1841–1852. 10.1016/j.susc.2008.08.037.

[ref30] Merino P.; Svec M.; Pinardi A. L.; Otero G.; Martin-Gago J. A. Strain-Driven Moire Superstructures of Epitaxial Graphene on Transition Metal Surfaces. ACS Nano 2011, 5, 5627–5634. 10.1021/nn201200j. 21675741

[ref31] Batzill M. The Surface Science of Graphene: Metal Interfaces, CVD Synthesis, Nanoribbons, Chemical Modifications, and Defects. Surf. Sci. Rep. 2012, 67, 83–115. 10.1016/j.surfrep.2011.12.001.

[ref32] Huttmann F.; Klar D.; Atodiresei N.; Schmitz-Antoniak C.; Smekhova A.; Martinez-Galera A. J.; Caciuc V.; Bihlmayer G.; Bluegel S.; Michely T.; Wende H. Magnetism in a Graphene-4 F-3d Hybrid System. Phys. Rev. B 2017, 95, 075427 10.1103/PhysRevB.95.075427.

[ref33] Martínez-Galera A. J.; Gómez-Rodríguez J. M. Influence of Metal Support in-Plane Symmetry on the Corrugation of Hexagonal Boron Nitride and Graphene Monolayers. Nano Research 2018, 11, 4643–4653. 10.1007/s12274-018-2045-5.

[ref34] Auwaerter W. Hexagonal Boron Nitride Monolayers on Metal Supports: Versatile Templates for Atoms, Molecules and Nanostructures. Surf. Sci. Rep. 2019, 74, 1–95. 10.1016/j.surfrep.2018.10.001.

[ref35] Martinez-Galera A. J.; Gomez-Rodriguez J. M. Structural and Electronic Properties of 3,4,9,10-Perylene Tetracarboxylic Dianhydride on H-Bn/Rh(110). J. Phys. Chem. C 2019, 123, 1866–1873. 10.1021/acs.jpcc.8b10810.

[ref36] N’Diaye A. T.; Bleikamp S.; Feibelman P. J.; Michely T. Two-Dimensional Ir Cluster Lattice on a Graphene Moire on Ir(111). Phys. Rev. Lett. 2006, 97, 215501 10.1103/PhysRevLett.97.215501. 17155746

[ref37] Donner K.; Jakob P. Structural Properties and Site Specific Interactions of Pt with the Graphene/Ru(0001) Moire Overlayer. J. Chem. Phys. 2009, 131, 164701 10.1063/1.3246166. 19894964

[ref38] N’Diaye A. T.; Gerber T.; Busse C.; Myslivecek J.; Coraux J.; Michely T. A Versatile Fabrication Method for Cluster Superlattices. New J. Phys. 2009, 11, 103045 10.1088/1367-2630/11/10/103045.

[ref39] Cavallin A.; Pozzo M.; Africh C.; Baraldi A.; Vesselli E.; Dri C.; Comelli G.; Larciprete R.; Lacovig P.; Lizzit S.; Alfe D. Local Electronic Structure and Density of Edge and Facet Atoms at Rh Nanoclusters Self-Assembled on a Graphene Template. ACS Nano 2012, 6, 3034–3043. 10.1021/nn300651s. 22404459

[ref40] Martinez-Galera A. J.; Brihuega I.; Gutierrez-Rubio A.; Stauber T.; Gomez-Rodriguez J. M. Towards Scalable Nano-Engineering of Graphene. Sci. Rep. 2014, 4, 7314 10.1038/srep07314. 25472802 PMC4255185

[ref41] Herbig C.; Knispel T.; Simon S.; Schroder U. A.; Martinez-Galera A. J.; Arman M. A.; Teichert C.; Knudsen J.; Krasheninnikov A. V.; Michely T. From Permeation to Cluster Arrays: Graphene on Ir(111) Exposed to Carbon Vapor. Nano Lett. 2017, 17, 3105–3112. 10.1021/acs.nanolett.7b00550. 28426934

[ref42] Martinez-Galera A. J.; Schroeder U. A.; Herbig C.; Arman M. A.; Knudsen J.; Michely T. Preventing Sintering of Nanoclusters on Graphene by Radical Adsorption. Nanoscale 2017, 9, 13618–13629. 10.1039/C7NR04491G. 28876003

[ref43] Will M.; Atodiresei N.; Caciuc V.; Valerius P.; Herbig C.; Michely T. A Monolayer of Hexagonal Boron Nitride on Ir(111) as a Template for Cluster Superlattices. ACS Nano 2018, 12, 6871–6880. 10.1021/acsnano.8b02127. 29920200

[ref44] Martinez-Galera A. J.; Gomez-Rodriguez J. M. Pseudo-Ordered Distribution of Ir Nanocrystals on H-Bn. Nanoscale 2019, 11, 2317–2325. 10.1039/C8NR08928K. 30662984

[ref45] Will M.; Hartl T.; de la Cruz V. B.; Lacovig P.; Lizzit S.; Knudsen J.; Michely T.; Bampoulis P. Growth, Stability, and Electronic Decoupling of Pt Clusters on H-Bn/Ir(111). J. Phys. Chem. C 2021, 125, 3880–3889. 10.1021/acs.jpcc.0c10136.

[ref46] Hartl T.; Herrmann D.; Will M.; Falke Y.; Grueneis A.; Michely T.; Bampoulis P. Silicon Cluster Arrays on the Monolayer of Hexagonal Boron Nitride on Ir(111). J. Phys. Chem. C 2022, 126, 6809–6814. 10.1021/acs.jpcc.2c00694.

[ref47] Martinez-Galera A. J.; Gomez-Rodriguez J. M. Nucleation and Growth of the Prototype Azabenzene 1,3,5-Triazine on Graphite Surfaces at Low Temperatures. J. Phys. Chem. C 2011, 115, 11089–11094. 10.1021/jp200613c.

[ref48] Horcas I.; Fernandez R.; Gomez-Rodriguez J. M.; Colchero J.; Gomez-Herrero J.; Baro A. M. Wsxm: A Software for Scanning Probe Microscopy and a Tool for Nanotechnology. Rev. Sci. Instrum. 2007, 78, 013705 10.1063/1.2432410. 17503926

[ref49] Goriachko A.; He Y.; Knapp M.; Over H.; Corso M.; Brugger T.; Berner S.; Osterwalder J.; Greber T. Self-Assembly of a Hexagonal Boron Nitride Nanomesh on Ru(0001). Langmuir 2007, 23, 2928–2931. 10.1021/la062990t. 17286422

[ref50] Carbogno C.; Gross A.; Meyer J.; Reuter K. O_2_ Adsorption Dynamics at Metal Surfaces: Non-Adiabatic Effects, Dissociation and Dissipation. In Dynamics of Gas-Surface Interactions; Springer: Heidelberg, 2013.

[ref51] Montemore M. M.; van Spronsen M. A.; Madix R. J.; Friend C. M. O_2_ Activation by Metal Surfaces: Implications for Bonding and Reactivity on Heterogeneous Catalysts. Chem. Rev. 2018, 118, 2816–2862. 10.1021/acs.chemrev.7b00217. 29116787

[ref52] Yadav G. D.; Mewada R. K.; Wagh D. P.; Manyar H. G. Advances and future trends in selective oxidation catalysis: a critical review. Catal. Sci. Technol. 2022, 12, 7245–7269. 10.1039/D2CY01322C.

[ref53] Valls Mascaró F.; McCrum I. T.; Koper M. T. M.; Rost M. J. Nucleation and Growth of Dendritic Islands during Platinum Oxidation-Reduction Cycling. J. Electrochem. Soc. 2022, 169, 112506 10.1149/1945-7111/ac9bdb.

[ref54] Rost M. J. Nucleation and Growth of Nano-Islands during Surface Reactions or Alloying with Increased Lattice Constant. J. Electrochem. Soc. 2023, 170, 012504 10.1149/1945-7111/acaa02.

[ref55] Rost M. J.; Jacobse L.; Koper M. Non-Random Island Nucleation in the Electrochemical Roughening on Pt(111). Angew. Chem., Int. Ed. 2023, 62, e202216376 10.1002/anie.202216376. 36821416

