# Supplementary material for: Unearthing Atomic Dynamics in Nanocatalysts
Source: ACS Appl Mater Interfaces. 2024 Oct 25;16(44):60348–55. doi: 10.1021/acsami.4c14382 (PMC11551902; doi:10.1021/acsami.4c14382)
Supplement: Supplementary file 1 — am4c14382_si_001.pdf [file am4c14382_si_001.pdf]

Supplementary Information

Unearthing atomic dynamics in nanocatalysts

Antonio J. Martínez-Galera[1,2,3*], *Rocío Molina-Motos*[1], *and José M. Gómez-Rodríguez*[2,3,4]

[1] *Departamento de Física de Materiales, Universidad Autónoma de Madrid, Madrid E-28049, Spain*

[2] *Condensed Matter Physics Center (IFIMAC), Universidad Autónoma de Madrid, Madrid E-28049, Spain*

[3] *Instituto Nicolás Cabrera, Universidad Autónoma de Madrid, Madrid E-28049, Spain*

[4] *Departamento de Física de la Materia Condensada, Universidad Autónoma de Madrid, Madrid E-28049, Spain*

S1. Nanoparticle growth over h-BN/Ru(0001) after partial Ir intercalation

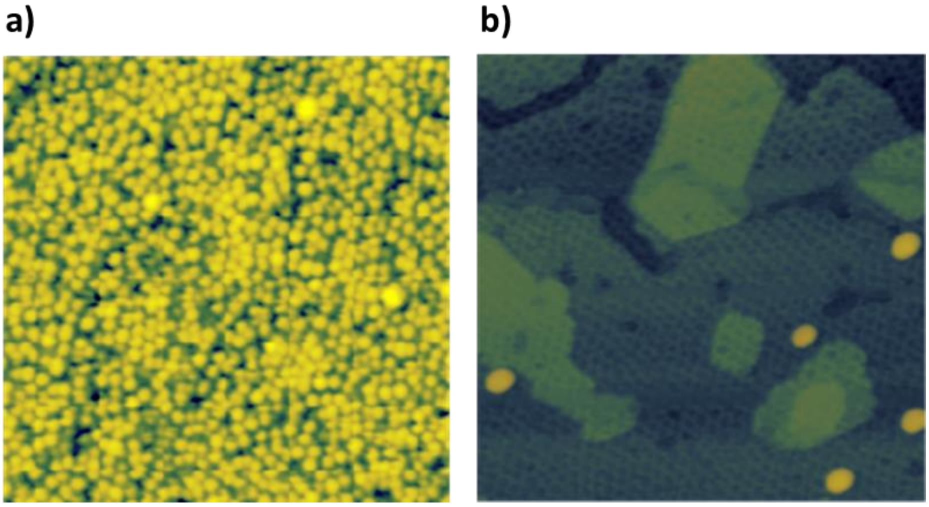

Figure S1. Ir nanoparticle growth on Ir intercalated h-BN/Ru(0001) surfaces and further Ir intercalation after annealing. **a)** Ir nanoparticle network grown after new 0.9 ± 0.1 ML Ir deposition on the sample acquired after the annealing sequence analyzed in Fig. 5 of the main manuscript. **b)** STM image acquired after annealing at 750 ºC during 3600 s the sample analyzed in a). Tunneling parameters: a) b) $V_s = +2.4$ V, $I_T = 0.35$ nA, size: 95 × 95 nm^2.

S2. Auger Electron Spectroscopy characterization of Ir nanoparticles grown on h-BN/Ru(0001) surfaces

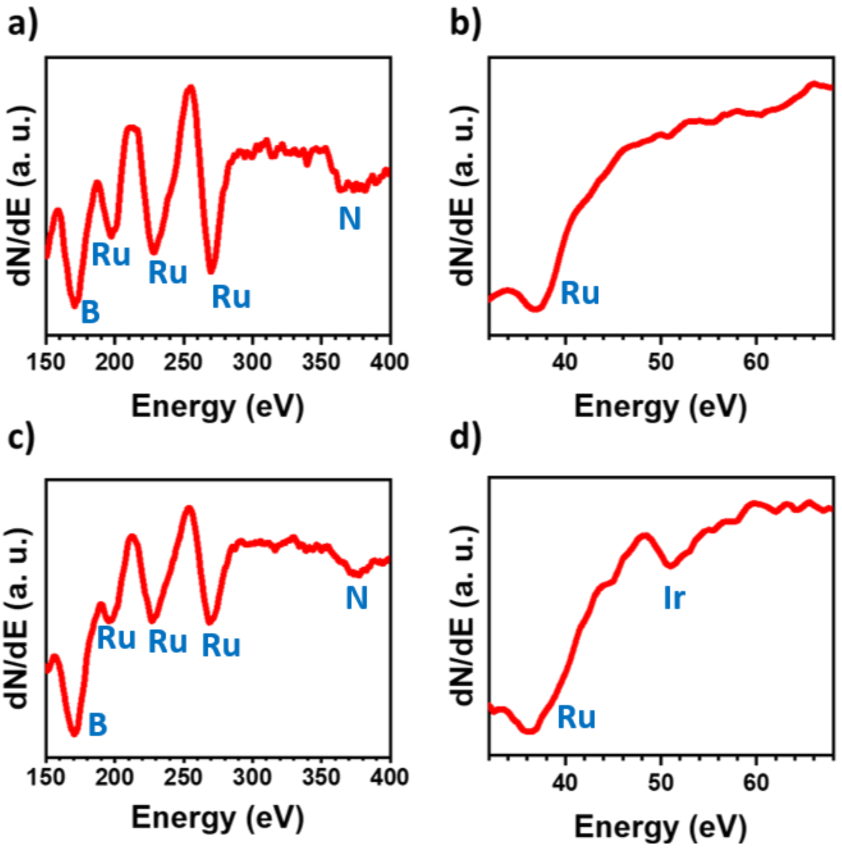

Figure S2. Auger Electron Spectroscopy (AES) study of Ir nanoparticles grown on h-BN/Ru(0001) surfaces. **a)-b)** AES spectra acquired on a pristine h-BN/Ru(0001) surface. **c)-d)** AES spectra acquired on an Ir nanoparticle network grown after [0.8 ± 0.2] ML Ir deposition on a h-BN/Ru(0001) surface.
